# The Effects of Biting and Pulling on the Forces Generated during Feeding in the Komodo Dragon (*Varanus komodoensis*)

**DOI:** 10.1371/journal.pone.0026226

**Published:** 2011-10-20

**Authors:** Domenic C. D'Amore, Karen Moreno, Colin R. McHenry, Stephen Wroe

**Affiliations:** 1 Graduate Program in Ecology and Evolution, Rutgers, The State University of New Jersey, New Brunswick, New Jersey, United States of America; 2 Natural Sciences Department, Daemen College, Amherst, New York, United States of America; 3 School of Biological, Earth and Environmental Sciences, University of New South Wales, Sydney, New South Wales, Australia; 4 Laboratorio de Paleontología, Universidad Austral de Chile, Valdivia, Chile; 5 Laboratoire de Anthropologie Moleculaire et Imagerie de Synthèse, Toulouse, France; 6 Géosciences Environnement Toulouse, Toulouse, France; 7 School of Engineering, University of Newcastle, Callaghan, New South Wales, Australia; 8 Department of Anatomy and Developmental Biology, Monash University, Victoria, Australia; University of Western Ontario, Canada

## Abstract

In addition to biting, it has been speculated that the forces resulting from pulling on food items may also contribute to feeding success in carnivorous vertebrates. We present an *in vivo* analysis of both bite and pulling forces in *Varanus komodoensis*, the Komodo dragon, to determine how they contribute to feeding behavior. Observations of cranial modeling and behavior suggest that *V. komodoensis* feeds using bite force supplemented by pulling in the caudal/ventrocaudal direction. We tested these observations using force gauges/transducers to measure biting and pulling forces. Maximum bite force correlates with both body mass and total body length, likely due to increased muscle mass. Individuals showed consistent behaviors when biting, including the typical medial-caudal head rotation. Pull force correlates best with total body length, longer limbs and larger postcranial motions. None of these forces correlated well with head dimensions. When pulling, *V. komodoensis* use neck and limb movements that are associated with increased caudal and ventral oriented force. Measured bite force in *Varanus komodoensis* is similar to several previous estimations based on 3D models, but is low for its body mass relative to other vertebrates. Pull force, especially in the ventrocaudal direction, would allow individuals to hunt and deflesh with high success without the need of strong jaw adductors. In future studies, pull forces need to be considered for a complete understanding of vertebrate carnivore feeding dynamics.

## Introduction

The force applied to food items by vertebrates is strongly associated with morphology, behavior, and fundamental ecological niche. Bite force, or the reaction force at some point(s) in the jaws generated by adductor muscles, has been estimated for a wide range of both extinct and extant taxa on the basis of both 2D and 3D cranial modeling [1]–[7], mandibular morphometrics [8], [9], body mass estimates [10], and tooth marks on bones [11]. *In vivo* data has also been collected from several live vertebrates [12]–[15]. Bite force has consequently been correlated to relative prey size [16], ontogeny [17], [18], sexual dimorphism [19], and trophic ecology [3], [5].

Less frequently considered is the role of pulling on the prey, presumably facilitated mostly by postcranial musculature, on feeding success. Muscles extrinsic to the jaw apparatus may play an important role in amplifying the forces applied to food items for many taxa [4], [6]. Several studies have directly observed feeding behaviors that incorporate postcranial muscles and some have modeled the forces [20], [21]. Although modeling approaches are useful in a comparative context they can considerably misrepresent actual forces [4], [22]. These forces are rarely measured *in vivo*. The most notable example is the quantification of rotational forces produced by postcranial muscles in caecilians to reduce oversized food items [23].

The morphology and behavior of *Varanus komodoensis*, the Komodo dragon, may best exemplify the potential significance of pulling on feeding success. The largest living lizard [24], [25], it has laterally flattened, curved, serrated tooth crowns categorized as ziphodont [26]. The cranium has a dorsoventrally flattened rostrum, and a relatively lightly-built construction consisting of minimized skeletal elements [27]. Finite element modeling approaches indicate *V. komodoensis* has a bite force that is relatively low for its body size, and muscle measurements indicate relatively small forces produced by the jaw adductor muscles [8], [27], [28]. The skull can resist large stresses in the lateral and caudal direction though, suggesting the weak jaw adduction force may be supplemented by pulling for effective hunting and defleshing.

*Varanus komodoensis* feeding behavior also suggests the importance of forces produced by muscles extrinsic to the jaw. As an ambush predator, it incorporates a slashing bite that causes major blood loss or evisceration [25]. This is coupled with venom secreted from glands in the mandible, which function both as a neurotoxin and an anticoagulant [28]. When defleshing a carcass, individuals rotate their heads so that the teeth cut in sequence along a curved line [25], [29]. This behavior rotates the head from a position lateral to the rest of the body to one more medial, while simultaneously moving caudally [30]. This medial-caudal arc is coupled with straightening of the forelimbs or back-pedaling, which draws the rostrum further in the caudal direction. This allows *V. komodoensis* to disarticulate the carcass, and swallow portions via inertial feeding [25], [31].

Force production in *V. komodoensis* is also relevant to the reconstruction of paleontological systems. The distinct *V. komodoensis* feeding methodology is the exception rather than the rule concerning modern day reptiles, and its cranio-dental condition does not occur in any other extant tetrapods [25]. Conversely, this morphological condition is similar to a large number of extinct taxa, especially to theropod dinosaurs. Conclusions about theropod feeding behavior are often made based on the basis of this qualitative comparison [32]–[34]. The ziphodont condition is often seen in extinct crocodylians as well [26]. *Varanus komodoensis* is also one of the last of the giant varanids to radiate during the Pliocene [35], and is a sister taxon to the extinct *V. prisca* (‘*Megalania*’), the largest know terrestrial lepidosaur [36].

Based on its morphology, behavior, and paleontological significance, *V. komodoensis* is an ideal study animal to quantitatively assess the significance of force generated by pulling relative to jaw adductor generated bite forces. In this study, bite force and pulling force is collected from captive *V. komodoensis* individuals using force gauges/transducers. The major purpose of this study is to determine to what degree pulling force contributes to feeding behavior relative to jaw adductor generated bite force. We determine whether these forces correlate with body mass and length and evaluate the data in reference to cranio-dental structure. Lastly, the data will be compared to those available for other extant vertebrates. Aside from a preliminary note in Moreno et al. [27], this is the first study to collect *in vivo* feeding forces from *V. komodoensis*. All previous quantifications to date are solely model based. This study quantitatively considers pulling forces and their role, which is especially important because it will indicate how musculature outside of the jaw adductors, especially postcranial musculature, can potentially influence the feeding success of a vertebrate predator.

## Methods

### Ethics Statement

Force data was derived from ten captive *V. komodoensis* individuals from four locations: Denver Zoo in Denver, CO, Miami Metro Zoo in Miami, FL, Lowry Park Zoo in Tampa, FL, and Disney's Wild Kingdom in Orlando, FL. All procedures concerning live animals were approved by their respective Internal Animal Care and Use Committee. All trails were supervised by a trained employee of their respective host institution. These procedures were also endorsed under the American Zoo and Aquarium Association's Species Survival Plan for *V. komodoensis*.

### Monitor Lizard Characters

All bite/pull force trials were recorded using a handheld video camera. Several morphological characters were noted (Table 1). Zoo staff usually documented age, sex, mass, and total body length (TL). The two oldest specimens were wild caught at an unknown age 20 years before data collection. Neck and limb measurements were not collected.

**Table 1:** Characteristics of *Varanus komodoensis* specimens used in this study.

Identification #	Location	Sex	Age (years)	Mass (kg)	TL (cm)	HL (cm)	HW (cm)
301734	Lowry Park Zoo	♂	13	60.00	228.60	19.29	13.11
940339	Denver Zoo	♂	12	50.20	244.00	19.22	12.06
981742	Disney World	♂	13	26.36	-	16.87	10.01
981745	Disney World	♀	14	25.45	-	17.54	11.76
98R046	Miami Metro Zoo	♀	8	28.18	177.8	15.75	10.54
98R068	Miami Metro Zoo	♀	8	25.45	172.72	15.03	9.89
98R069	Miami Metro Zoo	♂	8	36.81	187.96	16.43	10.77
H00957	Miami Metro Zoo	♂	>20	74.77	236.22	19.81	13.54
H00958	Miami Metro Zoo	♀	>20	47.27	185.42	17.53	13.51
R00026	Miami Metro Zoo	♂	14	55.45	-	-	-

TL = total body length; HL = head length; HW = head width.

Photographs taken with a mounted camera were taken of each individual's head from the dorsal perspective with a scale. All specimens were similarly aligned. Random landmarks were plotted outlining the head using the morphometric landmark software TpsDig2 [37]. The base of the head was easily identified by the contours of the head and scale morphology. These landmarks were standardized into 31 equidistant landmarks using Chainman 3D [38]. All of the landmarks directly opposite to one another were measured to determine which set was farthest apart. The distance between this specific set was determined to be the maximum width of the head (HW) for each individual. This was usually close to the base. The length of the head (HL) was calculated as the distance between the rostral most landmark (the 16^th^) and the midpoint between the two caudal landmarks.

### Force data collection

The transducer used to collect bite force values consists of two aluminum beams with Wheatstone bridge style strain gauges between them ([39]; see also [40], [41]). This particular transducer originally was constructed by Binder and Van Valkenburgh [17] to acquire bite force values from spotted hyenas (*Crocuta crocuta*). Monitors bit down on the ends of the beams, which were covered with rubber to prevent tooth damage. Deformation of the beams relays information in volts using Logger Pro 3 (Vernier Software and Technology), which is then converted to kilograms through calibration methods outlined in DeChow and Carlson [39] and subsequently converted to Newtons (N). During each trial, the bite force transducer was introduced to a single *V. komodoensis* individual in the enclosure where it was usually fed. To induce a biting response, a small strip of horse meat was fastened to the transducer using an elastic band. Once the individual removed the meat, the process was repeated. Between three to five trials were conducted with each *V. komodoensis*.

Pull force was recorded using a Chatillon® DFS series digital force gauge, and the output was processed using NEXYGEN DF series software. For pull strength, the carcasses secured to the force gauge were pork necks purchased from a local vendor. Each weighed approximately 0.75 kg, and consisted of mid-sagittally halved articulated cervical vertebrae and the cranial- and thoracic-most vertebrae and ribs, with the majority of the flesh removed. Carcasses were tied to the force gauge using a 1.6 mm crimped metal wire. Carcasses were introduced from two angles from the ground. The “low angle” gauge position placed the pull gauge near the ground with the carcass on the ground of the enclosure. The “high angle” gauge position placed the gauge approximately 1.5 m off the ground with the carcass suspended just above the ground. The height was chosen solely because of the size limitations of the certain enclosures. These gauge positions measured force from the caudal and ventrocaudal direction respectively. The ecological significance of the caudal pull could symbolize immobilized prey, or the force on a carcass that is being scavenged. The ventrocaudal pull is significant because it could represent a *V. komodoensis* pulling down live prey when hunting [26]. A total of two to four trials were conducted with each individual, each lasting 300 seconds.

Because of its uniaxial nature, the orientation of the gauge needed to be parallel to the direction of force exerted on it. Since the monitor lizards changed position frequently, the researcher held the gauge and manually changed its orientation by turning it to face the monitor throughout the trail. Measures were taken to minimize gauge countermovement produced by the researcher, so as to not inflate the data. For most trials the gauge was propped against either the enclosure walls/fencing or the ground. This held the gauge in place and offered resistance against the *V. komodoensis* individual's force. In some trails from the low angle introduction this was not possible, and the researcher had to hold the gauge stationary solely with their hands. The researchers seated themselves with their hands and arms firmly positioned against their body to minimize the amount of gauge countermovement.

### Force quantification and statistics

**Figure 1:**
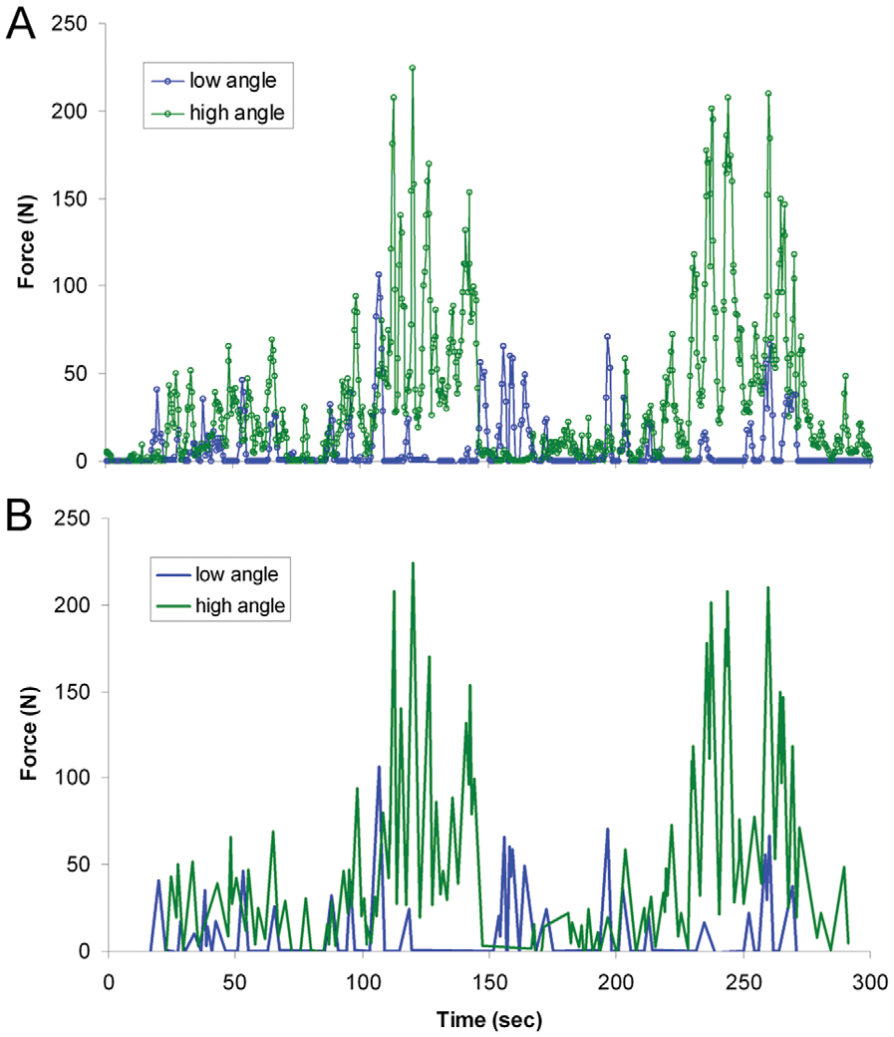
Typical high and low angle pull force data. (A) Actual data points collected from the force gauge; (B) data simplified into ‘peaks’. Data was from separate trials using *Varanus komodoensis* 301734.

**Figure 2:**
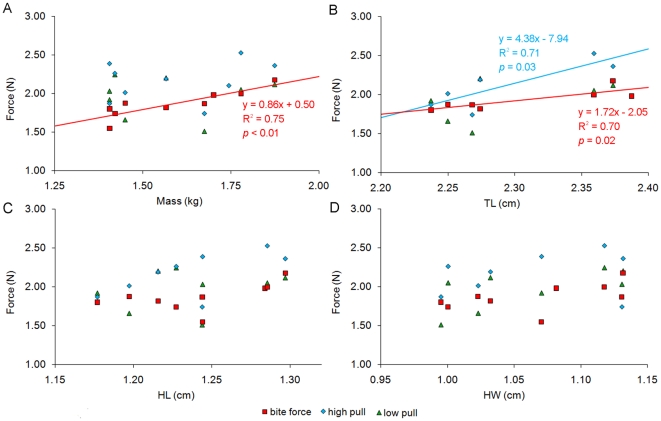
Force measurements in relationship to body size characters. Maximum force (N) per *Varanus komodoensis* individual versus mass (A), total body length (TL) (B), head length (HL) (C), and maximum head width (HW) (D). All data is log^10^−log^10^ transformed, and only significant regressions are displayed.

Using labVIEW, bites and pulls that generated more that 4N were isolated from the data into “peaks”. This boundary was arbitrarily selected because it eliminated an unmanageable amount of data that was insignificant to the goals of this study. Each peak is a discrete unit, representing a single force producing behavior. (For the output of a typical trail and how it is converted into peaks, see Figure 1). Data was log^10^−log^10^ transformed, and the maximum bite and pull forces for each *V. komodoensis* were plotted against the mass, TL, HL, and HW of each individual. Any significant regressions were plotted and elaborated upon (Figure 2).

## Results

### Bite force

When *V. komodoensis* bit the transducer, it remained engaged until the meat was removed. Individuals would follow biting with head movement solely in the lateral direction, with no rotation witnessed. Contact was rarely made between the transducer and the distal-half of the tooth row. A total of 200 bite force peaks were collected from 22 trials. Regressions indicate maximum bite force correlates best with mass, but also well with TL (Figure 2). All monitors produced a wide range of forces, but heavier monitors produced greater maximum forces with the highest at 148.56 N. HL and HW show much less significant correlations with bite force.

### High angle gauge position

**Figure 3:**
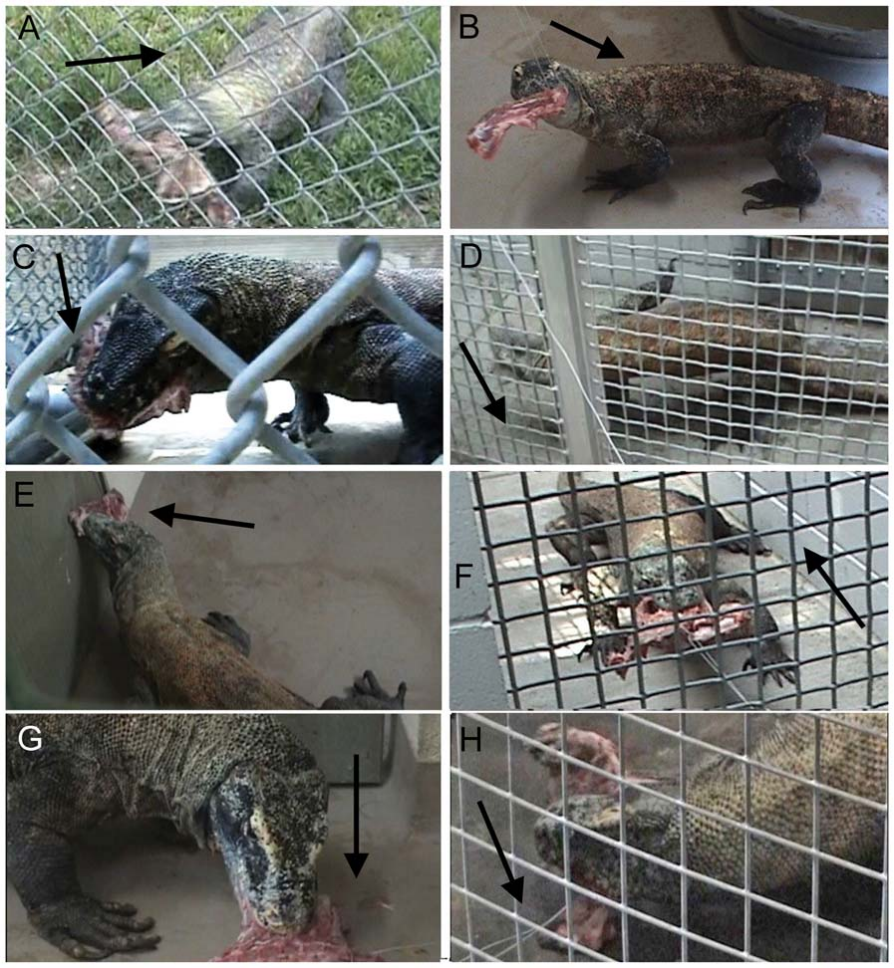
Behaviors witnessed during the pull force data collection. The arrows indicate the direction of pulling force. (A–E) represent high angle introduction, and (F–H) represent the low angle. (A) ventrocaudal pull with back-pedaling (98R046); (B) head lateroflexion (98R068); (C) head ventroflexion (98R069); (D) lifting the forelimbs/hind limbs off the ground (981745); (E) defleshing against the wall (H00957); (F) caudal pull with backpedaling (981745); (G) defleshing against the floor (H00958); (H) pressing the carcass against the floor (301734).

*Varanus komodoensis* pulls the carcass aggressively in the ventrocaudal direction from the high angle. Carcasses were first advanced into the mouth via inertial feeding. Gradual neck movements reoriented the head either ventrally or laterally. Repetitive cranial-caudal ‘rocking’ movement due to the straightening and bending of the forelimbs (witnessed by Burden [29]), lateral shaking, sudden caudal ‘jerks’ of the head and neck, and/or back-pedaling pulled the carcass caudal (Figure 3A–C). Some individuals would even appear to ‘hop’ because pulling downwards quickly on the taut wire lifted the limbs from the ground (Figure 3D). This yielded the largest force. Certain individuals would place a portion of flesh within their mouths, push the carcass in the cranial direction against the wall of the enclosure, and deflesh it incorporating slow, repetitive strokes (Figure 3E). These behaviors did not create significant pulling force.

A total of 739 peaks were produced from 17 high angle trials. The force applied during these trials was variable and had a wide range, with the greatest maximum force at 336.5 N (Figure 2). There was no significant correlation between mass and maximum pulling force. In fact, one of the lightest individuals (25.45 kg) produced the second highest maximum force (243.77 N). Only TL produced a regression that showed a significant positive correlation.

### Low angle gauge position

The low angle pull produced a total of 366 peaks from 14 trials; much less than in the high angle pull (Figure 1). Maximum force values were lower as well, with the greatest at 175.07 N. Several behaviors exhibited were similar to those displayed at the high angle. Monitors would pull their head and neck either caudally or laterally (Figure 3F), accompanied by rocking, quick jerks, and backpedaling. Unique to the low angle, many individuals defleshed the carcass with it stationary on the ground (Figure 3G), and all individuals would place the carcass in their mouth and push it against the ground to force it further down the gullet (as previously witnessed [25], [30]; Figure 3H). These behaviors did not make the wire taut, and significant force was not recorded due to the unidirectional nature of our gauge. Consequently, low pull was not significantly correlated with any of the four variables (Figure 2).

## Discussion

### Force production in *Varanus komodoensis*

Maximum bite force in *V. komodoensis* correlates best with body mass, as in other reptiles like xenosaurid lizards and crocodylians [12], [18]. This suggests that as the monitors gain more mass they accumulate more jaw adductor musculature. Older males were usually heaviest, and therefore had the highest forces. TL shows a degree of correlation with bite force as well, most likely because as the monitors grow in mass their length increases. HL and HW did not correlate well with bite force. It is particularly interesting that HW did not correlate well, because it was assumed that this width would be increased by enlarged muscles; especially the *m. pterygoideus*. Future studies should investigate more reliable, non-invasive methods for measuring adductor muscle mass on live specimens.

The *in vivo* bite force data largely support predictions made on the basis of 3D finite element modeling. A *V. komodoensis* individual modeled by Moreno et al. was predicted to have a maximum bite force of 10–20 N at sub-optimal gape [27] and 39 N at optimal gape [28]. When this individual's TL (160.00 cm) is incorporated into our best-fit regressions, it would bite at a maximum of 53.83 N. This indicates a maximum bite force only slightly greater than those predicted by modeling at optimal gape. We note, however, that the model-based estimations are from a bite point about halfway along the tooth-row, where as the *in vivo* bites were typically more anterior.

2D models have been less accurate in predicting *V. komodoensis* bite force. Using the Sinclair and Alexander beam approach [8], the individual modeled by Moreno et al. above would have an anterior bite force of 11 N (taken from Moreno et al. [27]). This greatly underestimates our *in vivo* results. Therrien et al. [9] also proposed that a *V. komodoensis* individual with a 16.96 cm mandible should have a bite force 0.086 times that of an *Alligator mississippiensis* individual with a mandible length of 50.08 cm. Using the regression derived from experimentally acquired *A. mississippiensis* bite forces from Erickson et al. [18], the *V. komodoensis* individual modeled by Moreno et al. (HL = 14.20) [27] would have a maximum bite force of 695.97 N, greatly overestimating its capability.

**Figure 4:**
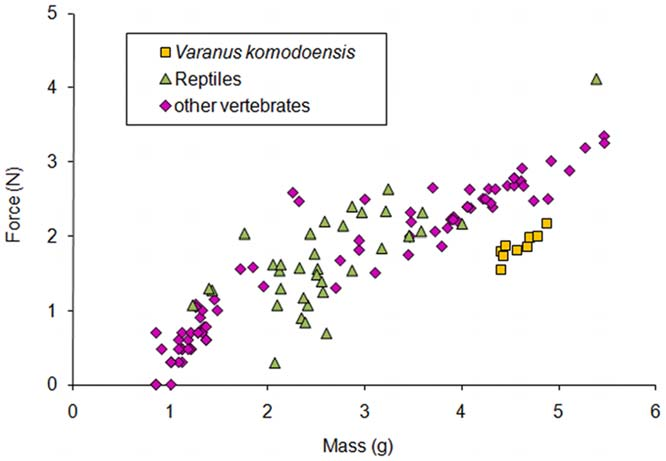
*Varanus komodoensis* bite force in relationship to extant taxa. (A) *V. komodoensis* plotted (log^10^−log^10^) against a range of previously collected vertebrate data taken from Huber et al. [15].

Maximum *V. komodoensis* bite forces are noticeably lower than those produced by other vertebrates with similar masses. Our *in vivo* data falls well below that of all the vertebrates of similar masses when plotted against Huber et al. 's [15] comprehensive list of anterior maximum bite forces (Figure 4). Even noticeably smaller reptiles have considerably larger maximum bite forces. Meers [10] proposed a function based on maximum bite forces of extant carnivores in relationship to mass (y = 0.92×+4.38). If our *V. komodoensis* masses were plotted along that same axis, the bite forces observed would be two orders of magnitude lower than what the function predicts. Anterior bite forces in *V. komodoensis* are also well below those estimated for mammalian carnivores of comparable body mass [16].

In the case of the *V. komodoensis*, pulling strength, especially in the ventrocaudal direction, was much larger than biting. Ventrocaudal force was generated predominantly by head ventroflexion, head lateroflexion, and caudal movement of the entire body. This body movement supports Moreno et al. 's assertion that muscles extrinsic to the jaw adductors, coupled with the total body mass of the individual, contribute the majority of force when feeding. Head ventroflexion during biting is seen in several other tetrapods that rely on it to increase bite force, including caecilians [23], [42] and skink lizards [43]. Ventrocaudally oriented force in *V. komodoensis* appears to be predicted by TL, suggesting body dimensions influence the generation of force. A longer body length may result in a longer neck and limbs, and their movement will pull carcass further from the gauge resulting in more tension. The two individuals who produced values that were positioned below the best fit trend happen to be the oldest monitors studied, and they did not display as aggressive behaviors as the others. This lack of ‘motivation’ may be a consequence the animal's age, or simply a consequence of the length of time they have been in captivity. It is possible these older animals are not as ‘excited’ about their food, and consequently do not display behaviors that produce great pulling forces as frequently as their younger counterparts.

Individuals generated significant force less frequently during the low angle gauge position, because the monitors often adopted feeding strategies resulting in forces that did not apply tension to the gauge. Although we predict that a significant amount of force was generated by these behaviors, it could not be quantified here.

It should be noted that the animals, being captive, most likely did not produce force equivalent to the maximum they are capable of physiologically. The forces produced by wild individuals of the same size would most likely be higher when used in a truly natural setting. This should be taken into consideration when determining how telling the maximum forces are here, as well as the degree to which they deviate from the models mentioned above.

### The *Varanus komodoensis* feeding method

*In vivo* data confirm previous assertions that *V. komodoensis* has a jaw adductor generated bite force that is surprisingly low for its size. When pull strength is included as a quantitative measure of feeding performance, bite force is markedly augmented. Caudal/ventrocaudal pulling is therefore essential for the modification of flesh, and adequate pull force is produced to achieve this. The skull is well-suited to withstand forces generated by pullback loading in the caudal direction [27]. The curved apices on *V. komodoensis* ziphodont teeth, combined with this caudal head movement, allows for initial, direct contact between the apices and flesh when biting. Lateral flattening and serrations allow for the teeth to further move through flesh after this initial puncture with relatively little resistance [44]. This methodology of modifying flesh is described by Frazzetta [45] as a “puncture cut,” and is also believed to be the feeding method of both elasmobranch sharks [45] and theropods [46].

The relative magnitude of biting and pulling forces contributes to the ability of to *V. komodoensis* to both hunt and deflesh carcasses. The generation of proportionately high ventrocaudally oriented forces may be a critical contribution to the ability of *V. komodoensis* to bring down prey larger than itself. When defleshing, repetitive punctures cut the flesh and eventually remove a portion to be swallowed [30]. This disarticulates the carcass as well; allowing the monitor lizard to swallow disarticulated skeletal elements resulting in relatively low wastage. There is consequently no need for strong jaw adductors to break bones for ingestion.

### Conclusions and future research

The disproportionately large forces resulting from pulling in the ventrocaudal direction provide an explanation as to how a carnivorous vertebrate can successfully secure and modify prey with a relatively low jaw adductor generated bite force. Jaw adductor force alone may not give a complete picture of the factors that contribute to feeding success. Future studies investigating feeding carnivore feeding mechanics should also consider forces outside of the jaw adductors before an appropriate model can be formulated.

The gauges used here were uniaxial, and force production is multidimensional by nature. In order to determine all the directions in which force is applied to a carcass, future studies should consider the three dimensional nature of *V. komodoensis* behavior. Using multiple cameras and a force platform would help achieve this goal. This will allow for a more quantitative approach that cannot be addressed using video alone. This will also help determine what muscles are used when applying force in certain directions. Detailed measurements of skull dimensions and musculature will indicate if muscle mass actually affects bite force, or if some other variable may be the cause. Other varanids should also be sampled to see if the forces observed are unique to the specialized feeding behavior of *V. komodoensis*, or if they are apparent in other varanids with different feeding strategies (for example: the durophagous *V. niloticus*).

Although the *V. komodoensis* behavior model is unique amongst extant taxa, it can shed light upon the feeding behaviors of extinct ziphodont tetrapods, especially theropods [34], [47]. Supposedly some theropods also had relatively low bite forces given their size and cranial morphology [1]. As in *V. komodoensis*, such low bite forces may have been supplemented by a strong pull and would not hinder the animal's ability to modify flesh. Both tooth mark data and cranial morphometrics suggest that theropods used caudally oriented force during feeding [47]–[49]. Modeled neck musculature implies that some theropods (i.e. *Ceratosaurus* and *Allosaurus*) also displayed significant ventroflexion, suggesting the “pulling” or “raking” of ziphodont teeth through the use of these postcranial muscles [50].
